# Inferring causal interplay between air pollution and meteorology

**DOI:** 10.3389/fdata.2025.1710462

**Published:** 2025-12-17

**Authors:** Yves Philippe Rybarczyk, Niralkumar Hemantbhai Dave, Tobias Isaac Tapia-Flores, Rasa Zalakeviciute

**Affiliations:** 1 School of Information and Engineering, Dalarna University, Falun, Sweden; 2 Biodiversidad, Medio Ambiente y Salud (BIOMAS), Universidad de Las Americas, Quito, Ecuador

**Keywords:** Convergent Cross Mapping (CCM), fine particulate matter (PM_2.5_), relative humidity (RH), nonlinear causality, urban climate dynamics

## Abstract

**Introduction:**

This study investigates the bidirectional causal interplay between PM_2.5_ and relative humidity (RH) in Quito, Ecuador. Focusing on a high-altitude city with complex terrain, the objective is to understand pollution-climate feedbacks over a two-decade span.

**Methods:**

The study employs Convergent Cross Mapping (CCM), a nonlinear empirical dynamic modeling approach. Hourly data were analyzed across four districts in Quito across two distinct time periods: 2004–2005 versus 2022–2024. Robustness of causality was confirmed using surrogate testing techniques.

**Results:**

The analysis reveals statistically significant, nonlinear, and time-variant couplings. While RH influenced PM_2.5_ in the early 2000s, the relationship inverted, with PM_2.5_ increasingly driving RH by the early 2020s. Partial-derivative analyses indicate shifting interaction signs and strengths. Notably, pollution was found to increasingly suppress RH, particularly in northern districts.

**Discussion:**

The observed suppression of RH by pollution is consistent with urban heat island amplification and radiative effects. These findings underscore the necessity of nonlinear causality frameworks for understanding environmental feedbacks in complex terrains. The study highlights the need for integrated air quality and climate strategies. Future research should expand variables and monitoring sites to further generalize these findings.

## 1 Introduction

Air pollution and climate change are among the most pressing environmental challenges of the 21^st^ century (World Meteorological Organization, 2022; Fuller et al., 2022). Their interdependence is increasingly recognized, particularly in urban environments where anthropogenic emissions and meteorological variability interact in complex ways (Intergovernmental Panel on Climate Change, 2022). Meteorological variables such as temperature, humidity, and wind speed influence the dispersion and transformation of pollutants, while pollutants like particulate matter (PM) and ozone can, in turn, affect atmospheric processes and climate dynamics (Fiore et al., 2015; EPA, 2024).

PM with aerodynamic diameter ≤2.5 μm (PM_2.5_) is a key pollutant linked to millions of premature deaths annually (State of Global Air, 2024). While most (99%) of the world's population is exposed to unhealthy air quality (World Health Organization, 2022), this burden is particularly severe in rapidly growing cities of the Global South, especially those in complex terrain (Molina et al., 2019; Hinestroza-Ramirez et al., 2023). Yet the dynamic interplay between meteorology and air pollution remains understudied in high-altitude urban environments, where topography and lower oxygen levels introduce additional complexity (Zalakeviciute et al., 2018a).

In the Andean Cordillera, Santiago de Chile has long faced high levels of air pollution, especially from PM_2.5_, worsened by surrounding mountains and frequent thermal inversions that trap pollutants (Tikoudis et al., 2022). Although stricter vehicle standards and improved public transport have reduced extreme pollution events, a significant portion of the population is still exposed to unhealthy levels of air pollution, particularly during winter inversions worsened by residential heating (Feron et al., 2023; Préndez et al., 2023).

Quito, the capital of Ecuador, presents a unique similar case for examining these interactions. Situated at 2,815 meters above sea level (ASL) in a narrow Andean valley, the city experiences frequent temperature inversions and stagnant air episodes that exacerbate pollution levels (Cazorla, 2016). The city's topography, combined with rapid urban growth and poor-quality fossil fuels have driven PM_2.5_ concentrations to exceed World Health Organization's (WHO) guidelines by a factor of three, despite various efforts to regulate traffic and fuel quality (Zalakeviciute et al., 2018b). Specifically, over the past two decades, diesel and gasoline improved, going from pre-Euro standards to Euro 2–3, which helped sustain the PM_2.5_ concentrations around national air quality standards (i.e., 15 μg/m^3^). Simultaneously, Quito is already experiencing impacts of climate change, including rising temperatures and increased wildfire risk (Zalakeviciute et al., 2025).

Understanding the causal dynamics between air pollution and meteorology in such a unique setting is critical for both scientific advancement and policy development. Traditional linear methods such as correlation and Granger causality have limitations in detecting true causal relationships in environmental systems (Sugihara et al., 2012; Runge et al., 2019). Correlation is symmetrical and cannot infer directionality, while Granger causality assumes linearity and stationarity; assumptions that are often violated in real-world atmospheric systems. Empirical Dynamic Modeling (EDM) and its extension, Convergent Cross Mapping (CCM), offer a more robust framework for causal inference in nonlinear, state-dependent systems. CCM reconstructs the state space of a system from time series data and tests whether one variable can predict another, thereby inferring causality (Yuan and Shou, 2022). It is particularly effective in detecting bidirectional and nonlinear relationships and has been successfully applied in recent air quality studies (Xia et al., 2022; Istiana et al., 2023; Chen et al., 2023; Rybarczyk et al., 2024, 2025; Zhou C. et al., 2025).

CCM is uniquely suited for the Quito context due to its ability to handle short, noisy, and nonlinear time series data. It can detect feedback loops and is resilient to false positives caused by shared seasonality. In previous studies, CCM has revealed causal relationships between PM_2.5_ and urban climate that were invisible to correlation-based methods (Istiana et al., 2023; Rybarczyk et al., 2025). Given Quito's complex topography, evolving emissions profile, and data limitations, CCM provides a scientifically rigorous and empirically validated approach for uncovering the true dynamics between air pollution and meteorology.

Despite growing recognition of the feedback between air pollution and climate, most studies have focused on how meteorology affects pollution, with limited attention to the reverse (Chen et al., 2018; Zheng et al., 2025). This imbalance is particularly pronounced in high-altitude cities like Quito, where unique topographical and climatic conditions may amplify pollution-driven meteorological effects. Moreover, the reliance on linear models in much of the existing literature limits our understanding of these complex interactions. For these reasons, this study aims to address the following two core questions:

- RQ1: Does air pollution influence local meteorological conditions in Quito?
- RQ2: Have the causal relationships between air pollution and meteorology changed over the past two decades?

To answer these questions, we apply CCM to a 20-year time series of hourly PM_2.5_ and relative humidity (RH) data from four districts in Quito, to (i) detect causal relationships, (ii) analyze the sign and strength of causality between PM_2.5_ and RH, and (iii) evaluate temporal changes in these causal relationships, in light of fuel and traffic regulations applied by the Ecuadorian policy makers.

The selection of RH as the primary meteorological variable is motivated by a previous study showing that the relationship between atmospheric moisture and PM_2.5_ is not simple or linear (Zalakeviciute et al., 2018a). That earlier work shows that RH can sometimes relate to pollution increases (e.g., in high-traffic areas), while at other times it is associated with removal processes (such as wet deposition). This established complexity makes RH an ideal candidate for advanced analysis of the causal relationship between a meteorological parameter and an air pollutant. The fact that the RH–PM_2.5_ interaction is known to be subtle, region-specific, and highly state-dependent creates the precise nonlinear dynamic system that CCM is designed to investigate.

Despite growing evidence of PM_2.5_-RH interplays, a comprehensive understanding of their dynamic causal relationships, particularly in highly dynamic urban environments like Quito, remains elusive. Existing research often relies on correlation-based methods or limited temporal scopes, leaving a significant gap in our ability to discern directional causality and its evolution over time. This study addresses this critical research gap by employing advanced CCM on long-term, high-resolution datasets from Quito, thereby offering a unique contribution: a robust, causality-driven assessment of how PM_2.5_ and RH influence each other, and crucially, how these causal links have shifted between the early 2000s and the 2020s amidst significant climate and urban development.

## 2 Materials and methods

### 2.1 Study site

**Figure 1:**
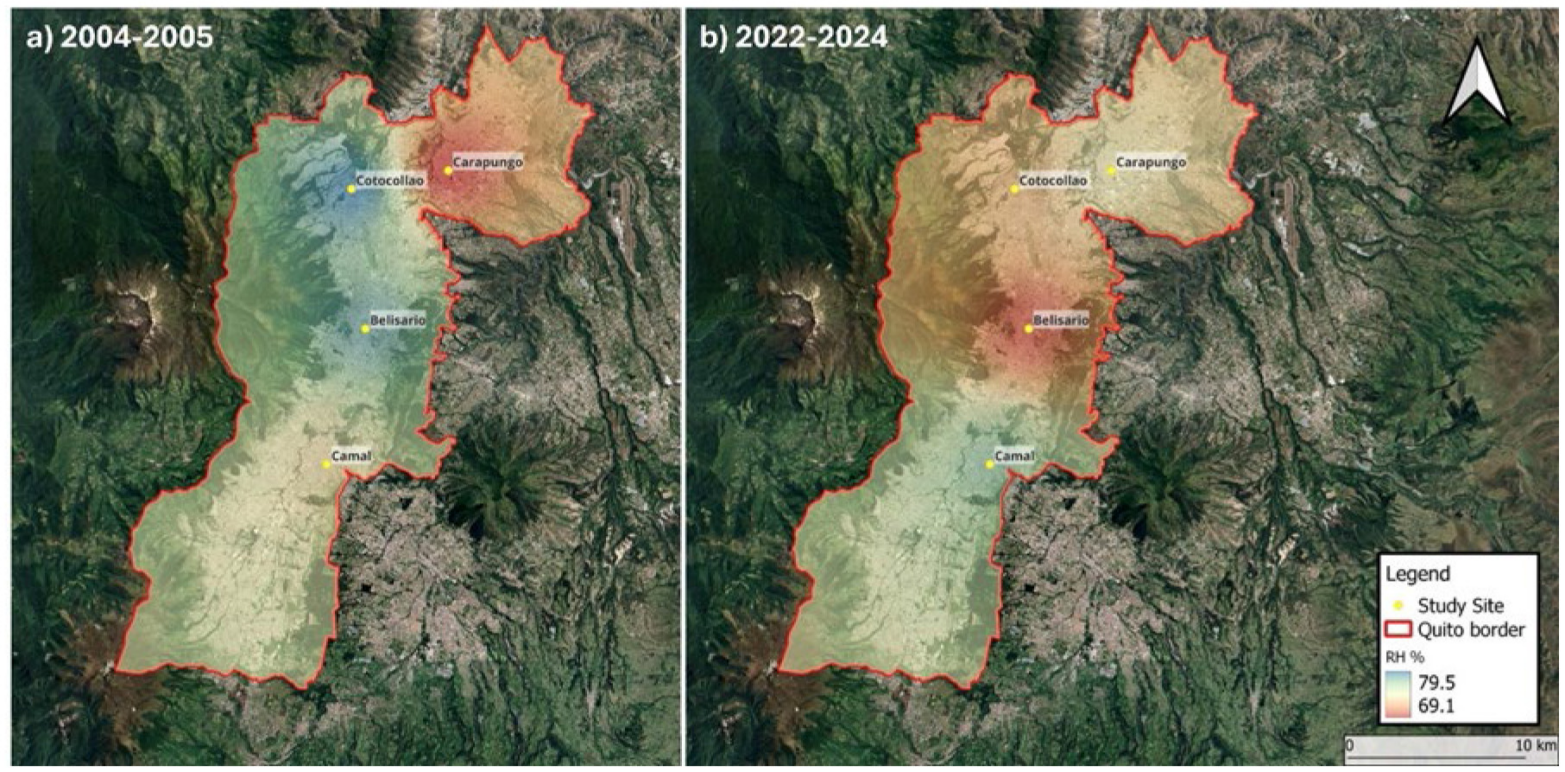
Delimitation of the urban district of Quito and location of the four study areas. A color gradient illustrates the percentage of relative humidity in the past **(a)** and the present **(b)**.

Quito, the capital of Ecuador, is located in the northern highlands of the country, nestled within the Guayllabamba River basin on the eastern slopes of the Pichincha volcano. At an elevation of approximately 2,850 meters above sea level, Quito is the highest constitutional capital in the world. The city's topography, characterized by an elongated inter-Andean valley bordered by the Andes Cordilleras, contributes to complex microclimates and significant air quality challenges (Zalakeviciute et al., 2018a). Low wind speeds and valley-induced stagnation often lead to pollutant accumulation, particularly from vehicular and industrial sources (Mancheno and Jorquera, 2025). Four districts were considered for this study: Belisario (2835 m ASL, coord. 78°29′24^′′^ W, 0°10′48^′′^ S), Cotocollao (2739 m ASL, coord. 78°29′50^′′^ W, 0°6′28^′′^ S), Carapungo (2660 m ASL, coord. 78°26′50^′′^ W, 0°5′54^′′^ S), and El Camal (2840 m ASL, coord. 78°30′36^′′^ W, 0°15′00^′′^ S) (Figure 1). These urban districts cover both urban and industrial areas and provide a representative characteristic of the air quality and local meteorology of the city.

### 2.2 Data collection and preprocessing

#### 2.2.1 Data sources

Data were obtained from the Secretariat of Environment under the Mayor's Office of Quito, which manages the city's air quality monitoring network. A complete atmospheric network consists of several ground stations with hourly observations from 2004 to 2024. Atmospheric sampling inlets are approximately 4 m above ground level, in accordance with U.S. EPA criteria for population-exposure monitoring (U.S. Environmental Protection Agency, 2024). Meteorological and air pollution sensors are co-located at the same height, as recommended for integrated urban air quality and meteorology networks to ensure consistency between pollutant and meteorological measurements.

This study focuses on PM_2.5_ and RH due to their relevance for analyzing the interaction between air pollution and climate change and for data quality reasons (i.e., less missing data). To quantify PM_2_.5 concentrations Beta Attenuation Monitor (Model FH62C14-DHS 5014i; EPA No. EQPM-0609-183) was employed. RH was measured using a Thies Clima Hygro-Thermo Transmitter compact sensor (Model 1.1005.54.161; Adolf Thies GmbH & Co. KG, Göttingen, Germany). The collected data are stored in the municipal administration's repository and are available for download at https://datosambiente.quito.gob.ec/ (accessed on 20 May 2025).

#### 2.2.2 Data cleaning and preparation

A structured preprocessing pipeline was implemented using R and Python. The percentage of missing data per monitoring station was 8.1% at Belisario, 6.7% at Carapungo, 7.7% at Cotocollao, and 3.6% at El Camal. Missing values were addressed using mean imputation for short gaps and forward-backward hill interpolation for longer gaps. Outliers were retained to preserve environmental variability. The final dataset included 100-day windows of hourly data for each district in two time periods: early 2000s (past period) and early 2020s (present period). This dataset was extracted from a temporal window of 2 years for the past period (2004-2005) and 3 years for the present period (2022-2024) to obtain a sample with the least amount of missing data for each site. The dataset's size balanced computational cost with temporal representativeness for nonlinear analysis.

The selection of 100-day windows based on data completeness was a necessary methodological trade-off. This approach ensures a high-quality, contiguous dataset essential for the robust application of CCM, which is well-suited to handling “short and nonlinear time series data”. However, this selection criterion introduces a potential sampling bias. By prioritizing the “cleanest” data, the chosen windows may not fully represent the complete seasonal dynamics of a given year, potentially skewing the analysis toward periods with fewer anomalous events (e.g., wildfires, extreme pollution episodes) if those events coincided with monitoring downtime or data gaps. While the retention of outliers within the window aimed to preserve variability, this limitation is acknowledged, and findings should be interpreted within this context.

### 2.3 Analytical methods

#### 2.3.1 Convergent Cross Mapping

CCM is a nonlinear empirical dynamic modeling technique used to detect causality in complex systems. It is based on State Space Reconstruction (SSR), which reconstructs the system's dynamics from time-lagged observations of a single variable. The principle of this method is that a variable X causes Y if the reconstructed state space of Y contains information about X. CCM tests this hypothesis by estimating X from Y's state space and measuring prediction or cross-map skill (*ρ*).

**Figure 2:**
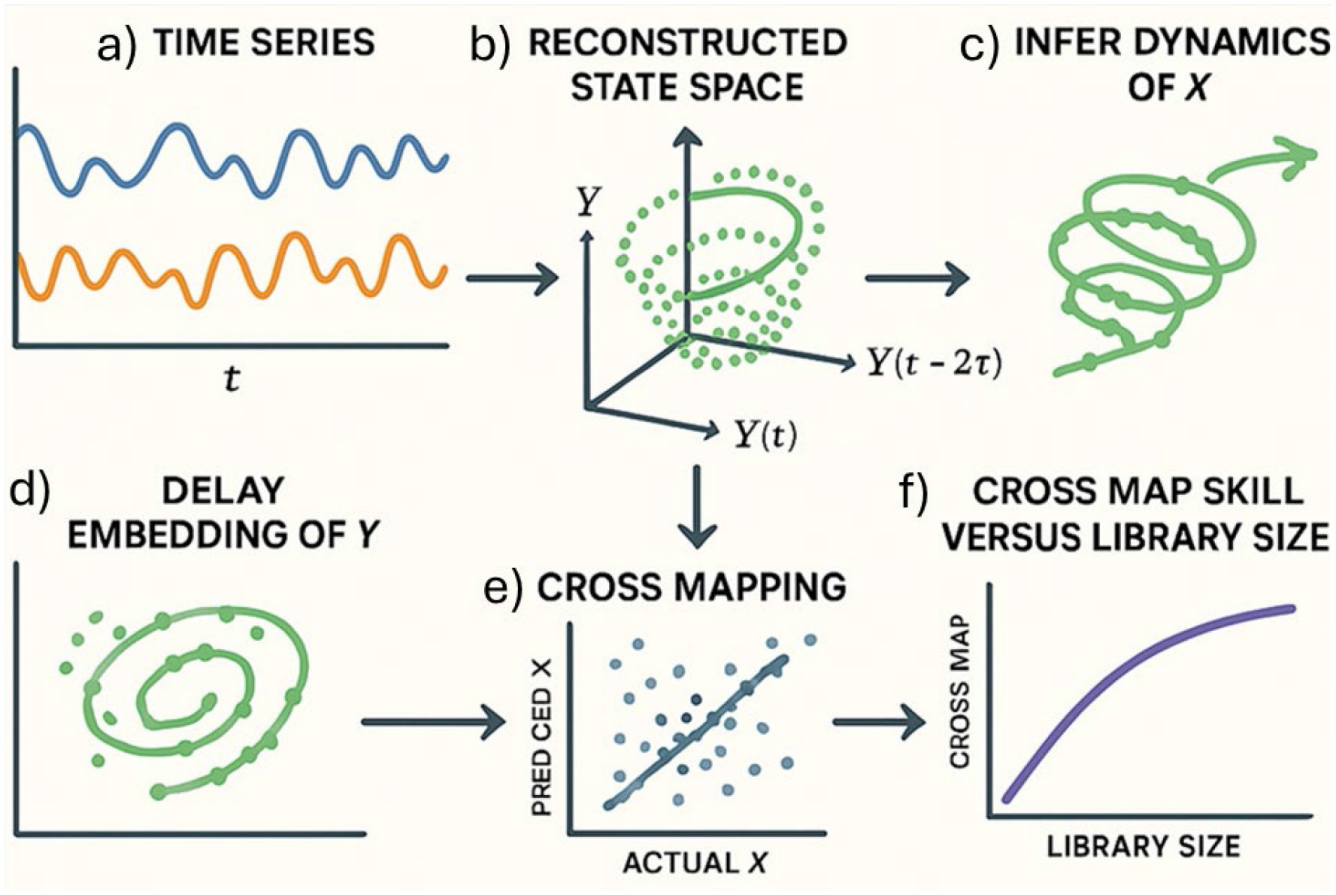
Illustration of the successive steps to proceed with the State Space Reconstruction (SSR) and the Convergent Cross Mapping (CCM). **(a)** Time series input: variables X (blue) and Y (orange) represent observed time series from a dynamic system. **(b)** State space reconstruction: using delay embedding, the state space of Y (green) is reconstructed, capturing its underlying dynamics. **(c)** Causal inference: if X influences Y, then the reconstructed state space of Y contains information about X. **(d)** Delay embedding: reconstruction of the state space of Y using time-delayed coordinates. **(e)** Cross mapping: the geometry of Y's state space is used to estimate values of X. The prediction accuracy is assessed by comparing the predicted X values with actual X values. **(f)** Library size: as more data is used (larger library), the prediction of X from Y improves if Y contains causal information about X.

As represented in Figure 2, the CCM procedure involves: (1) reconstructing the state space from time series data, using optimal embedding dimension (E) and time lag (*τ*), (2) identifying nearest neighbors in the state space, (3) estimating the driver variable using weighted averages, and (4) assessing convergence of prediction skill with increasing library size. A positive and converging *ρ* indicates causality.

#### 2.3.2 Implementation and parameter selection

CCM was implemented using the rEDM package in R. The reliability and interpretability of causal inference using CCM are highly dependent on the careful selection of key parameters: the embedding dimension (E) and the time lag (*τ*). These parameters define the reconstructed state space, which is fundamental to capturing the underlying dynamics of the system.

**Figure A.1:**
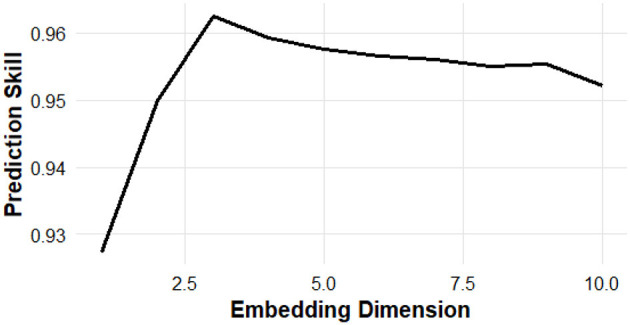
A graph illustrating the relationship between model prediction skill (y-axis) and embedding dimension (x-axis). The forecast skill peaks at E = 3 indicates that the dynamics of our data are unfolded best in 3 dimensions.

The embedding dimension (E) dictates the number of past states used to reconstruct the phase space. An insufficient can lead to a folded or degenerate attractor, obscuring true causal links by failing to unfold the system's dynamics adequately. Conversely, an excessively large can introduce noise and computational inefficiency without providing additional clarity, potentially diluting genuine causal signals. In this study, E was determined using simplex projection, where the optimal minimizes prediction error, ensuring the reconstructed attractor accurately reflects the system's dimensionality (Sugihara et al., 2012). The optimal embedding dimension was determined to be E = 3 (see Appendix Figure A.1, for a visualization of prediction skill against embedding dimension). Sensitivity analyses were performed around the optimal to confirm the robustness of the causal inferences and ensure that minor deviations did not qualitatively alter the findings.

The time lag (*τ*) specifies the interval between successive points used to construct the state vectors in the phase space. An inappropriate *τ* can lead to either highly correlated (redundant) or uncorrelated (noisy) coordinates, both of which hinder the effective reconstruction of the system's attractor. Typically, *τ* is chosen to be the first minimum of the average mutual information function, which identifies the time lag where new information about the system's state is maximized while redundancy is minimized (Fraser and Swinney, 1986). For the hourly PM_2.5_ and RH data, *τ* was often found to be around 12 hours, reflecting diurnal patterns in the atmospheric processes. Deviations from this optimal were also explored to verify that the identified causal relationships remained consistent within a reasonable range, thereby reinforcing confidence in the results' stability against minor parameter variations. The convergence of cross-map skill with increasing library size (L) served as the primary indicator of robust causality, and this convergence pattern was consistently observed across the range of optimally selected and values.

Once parameter selection was completed, nonlinearity was assessed using S-map analysis to confirm that CCM was suitable for the used dataset.

#### 2.3.3 Validation procedures

**Figure 3:**
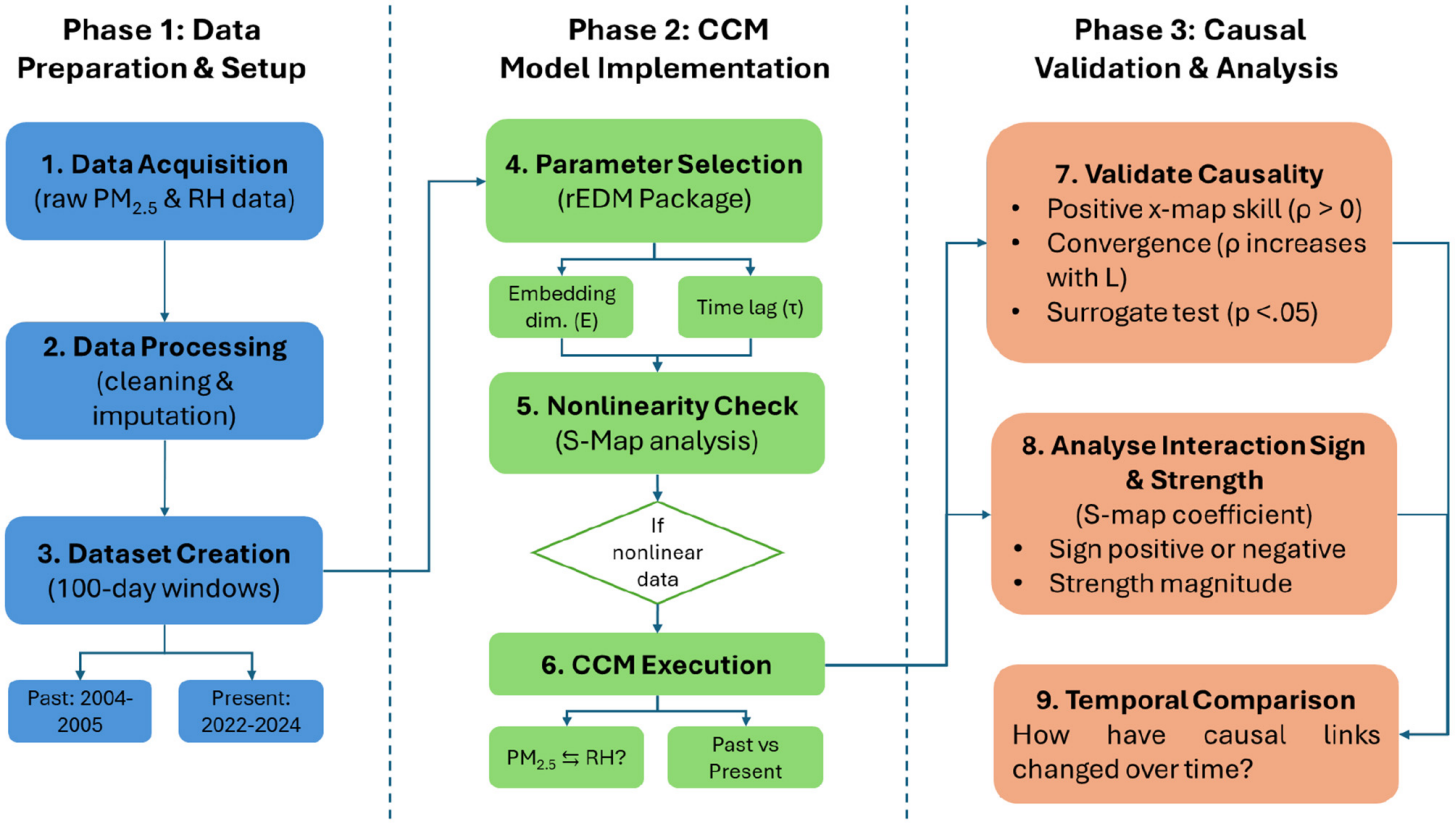
Workflow diagram of the CCM-based causal inference analysis.

At least three criteria must be satisfied to infer a causal relationship in the dynamic system. First, the cross-map (or x-map) skill must be positive. Second, the x-map should increase and converge with the library size (i.e., the amount of data). Third, the surrogate test must be significant. The Ebisuzaki method was employed to generate phase-randomized time series and assess the statistical significance of *ρ* (Ebisuzaki, 1997). Additionally, the sign of the state-dependent interactions was evaluated by computing the S-map coefficient, which measures the time-varying inter-variable interaction strength estimated by the S-mapping method as a partial derivative (Chang et al., 2017). These procedures ensured robustness against spurious associations to confirm the presence of genuine causal relationships. Figure 3 represents the full analytical pipeline applied in this study.

## 3 Results

This section presents the results of the Convergent Cross Mapping (CCM) analysis conducted to investigate the causal relationship between fine particulate matter (PM_2.5_) and relative humidity (RH) across four districts in Quito: Belisario, Cotocollao, Carapungo, and El Camal. The analysis compares two distinct time periods—early 2000s and early 2020s using convergence plots, surrogate significance tests, and partial derivative analysis to assess bidirectional causality, sign and strength of the causations, and their temporal evolution.

### 3.1 Convergent Cross Mapping

**Figure 4:**
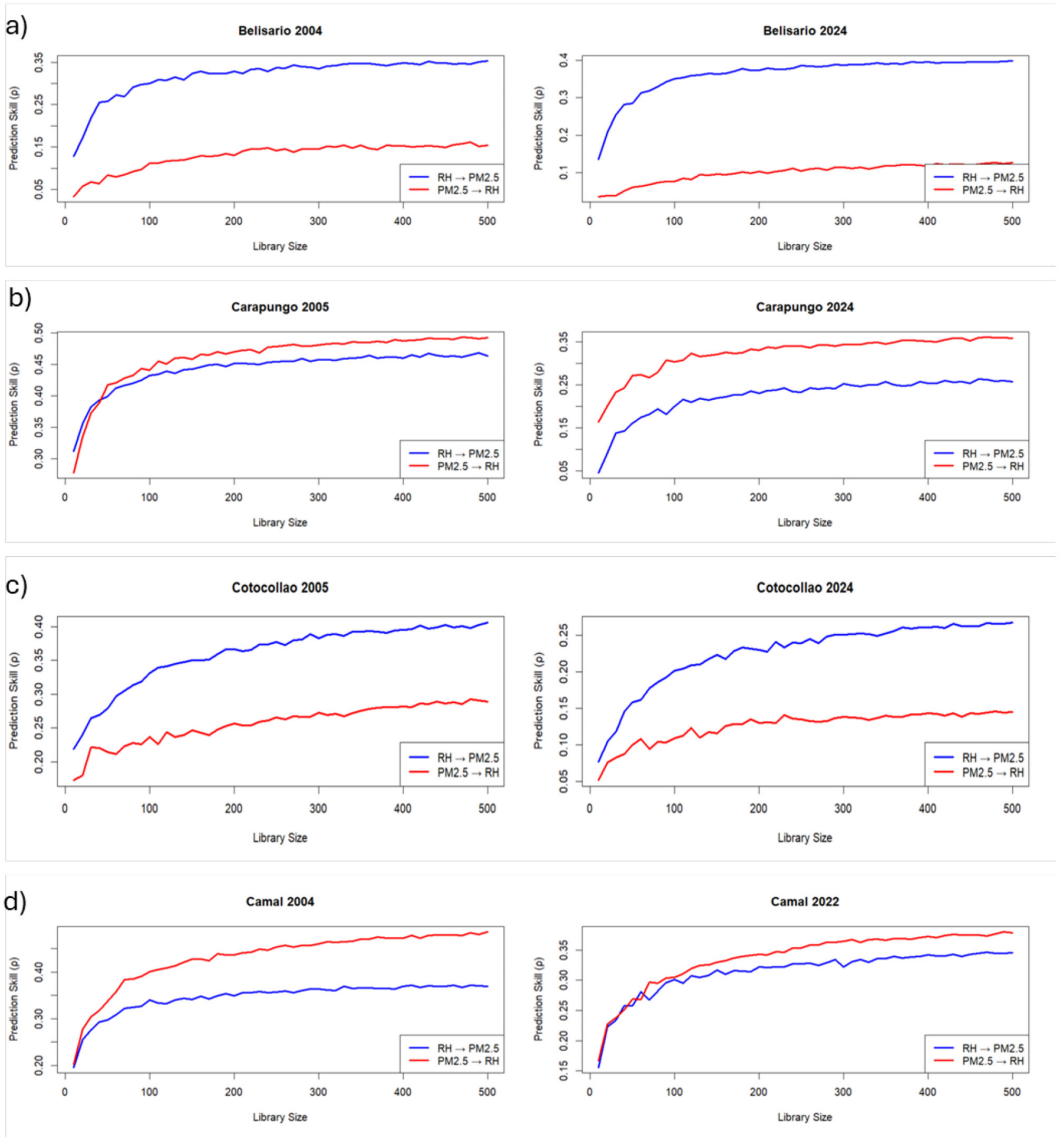
CCM results for the causal analysis between relative humidity (RH) and fine particulate matter (PM_2.5_) concentrations across the four study sites **(a–d)** and two periods: past (left column) vs. present (right column).

CCM analysis was applied to each district for two time periods. The x-map skill (*ρ*) was used to identify a possible causal interplay between fine particulate matter (PM_2.5_) and relative humidity (RH) in both directions. In Figure 4, RH → PM_2.5_ stands for a causal impact of RH on the concentration of PM_2.5_ in the atmosphere, and PM_2.5_ → RH stands for the effect of PM_2.5_ on the percentage of RH.

For RH → PM_2.5_, the x-map skill is positive and increases with library size across the four sites. This pattern persists throughout the two decades covered by the study. This result indicates that RH influences PM_2.5_ concentration. For PM_2.5_ → RH, the x-map skill is also positive and convergent for all sites and study periods, although *ρ* tends to be lower for this latter causal direction. This second observation implies a causal influence of PM_2.5_ concentration on the RH percentage across the entire city. Overall, these findings suggest a bidirectional causal effect between air pollution and urban climate, occurring at least over the past two decades. The mean x-map skill values across districts for both periods (2004–2005 and 2022–2024) is available in Appendix Table A.1.

### 3.2 Surrogate testing

To validate the CCM results, surrogate significance tests were conducted using 1,000 randomized time series for each direction. The original CCM skill was compared to the 95^th^ percentile of the surrogate distribution (Table 1). In all cases, the observed *ρ* values exceeded the surrogate thresholds (*p* < 0.05), confirming that the causal relationships are statistically significant and not due to shared seasonality or random fluctuations.

**Table 1:** Results of the surrogate tests for the four districts periods.

**Location**	**Year**	**Direction**	**Original*ρ***	**95^th^ Percentile**	***p*-value**
Belisario	2004	PM_2.5_ → RH	0.173	0.045	0.000
		RH → PM_2.5_	0.338	0.042	0.000
	2024	PM_2.5_ → RH	0.092	0.041	0.001
		RH → PM_2.5_	0.393	0.036	0.000
Cotocollao	2005	PM_2.5_ → RH	0.276	0.075	0.000
		RH → PM_2.5_	0.431	0.057	0.000
	2024	PM_2.5_ → RH	0.127	0.037	0.000
		RH → PM_2.5_	0.263	0.033	0.000
Camal	2004	PM_2.5_ → RH	0.486	0.044	0.000
		RH → PM_2.5_	0.347	0.042	0.000
	2022	PM_2.5_ → RH	0.374	0.041	0.000
		RH → PM_2.5_	0.340	0.043	0.000
Carapungo	2005	PM_2.5_ → RH	0.481	0.047	0.000
		RH → PM_2.5_	0.473	0.051	0.000
	2024	PM_2.5_ → RH	0.385	0.051	0.000
		RH → PM_2.5_	0.264	0.043	0.000

### 3.3 Sign and strength

**Figure 5:**
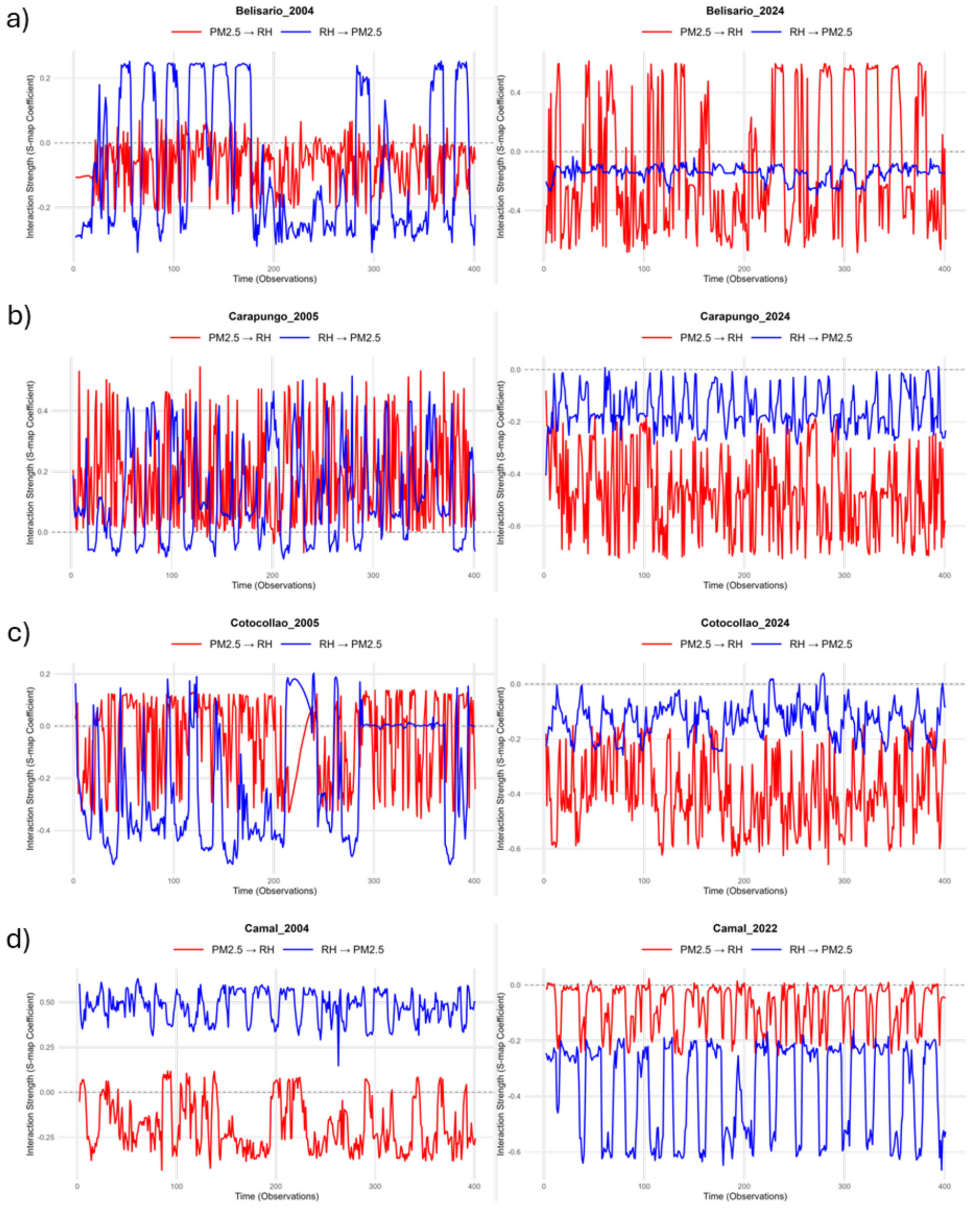
Partial derivative analysis to show the sign of the interaction strength between relative humidity (RH) and fine particulate matter (PM_2.5_) concentrations across the four study sites **(a–d)** and two periods: past (left column) vs. present (right column). A positive ∂ indicates that an increase in one variable induces an increase in the other variable (reinforcement effect). A negative ∂ means that an increase in one variable produces a decrease in the other variable (suppression effect).

Partial Derivative (∂) analysis was employed to investigate the state-dependent nature of causal relationships (Figure 5). The sign and strength of ∂ indicate the nature of the interaction. A positive ∂ signifies a reinforcing effect (e.g., an increase in PM_2.5_ leading to an increase in RH), while a negative ∂ indicates a suppressive effect (e.g., an increase in PM_2.5_ leading to a decrease in RH). This physically translates to whether the causal driver enhances or diminishes the response variable. The larger the magnitude of ∂, the stronger the influence of one environmental parameter on another.

In the early 2000s, the causal direction from RH to PM_2.5_ is more variable and stronger than that from PM_2.5_ to RH. The ∂ oscillation amplitude of RH to PM_2.5_ is approximately 0.5 for the northern (Carapungo and Cotocollao) and central (Belisario) districts. The sign of the effect varies from one site to another, oscillating between positive and negative values for Belisario, suggesting that humidity alternately promoted and inhibited particle concentrations (Figure 5a). An extended period of sustained negative interaction between observation points 180 and 280 indicates that humidity frequently suppressed particle concentrations during certain states, reflecting the complex nonlinear dynamics of urban atmospheric systems. For Carapungo, RH increases PM_2.5_ concentration (Figure 5b), while for Cotocollao, RH decreases PM_2.5_ (Figure 5c). El Camal exhibits slightly different behavior, showing a relatively constant and high positive effect on PM_2.5_ (∂ ≈ 0.5). By the 2020s, the influence of RH on PM_2.5_ diminishes. The amplitude of the ∂ does not exceed 0.2 for the northern and central parts of the city, and RH tends systematically to reduce PM_2.5_ concentration. The strongest negative effect of RH on PM_2.5_ is observed in the south (El Camal), where ∂ ranges between−0.2 and−0.6 (Figure 5d).

PM2.5 → RH evolves quite differently than RH → PM2.5 over the last two decades. PM2.5 → RH becomes more pronounced and negative, particularly in Belisario and Carapungo, indicating a shift toward pollution-driven suppression of RH. In Belisario, there is a drastic increase in ∂ oscillation amplitude from 2004 (−0.2 ≤ ∂ ≤ 0) to 2024 (−0.5 ≤ ∂ ≤ 0.5), suggesting that RH is strongly modulated by PM2.5 by the 2020s, while the effect of RH on PM2.5 is weaker (Figure 5a). In Carapungo, the impact of PM2.5 on RH reverses from 2005 to 2024. Figure 5b illustrates that air pollution is now reducing the percentage of RH in the atmosphere. Cotocollao, the second district in the north, exhibits a similar pattern, with an increasing negative effect of PM2.5 on RH from 2005 to 2024 (Figure 5c). In the case of El Camal, PM2.5 → RH maintains a relatively constant and moderate negative effect between 2004 and 2022 (Figure 5d).

These results align with the expansion of dry areas covering the city over the last two decades. In Figure 1, we can observe that the low RH areas were limited to the district of Carapungo for 2004–2005. By 2022–2024, the dry area has expanded to include both northern districts (Carapungo and Cotocollao) and the city center (Belisario). Only El Camal remains with high RH and a moderate effect of PM2.5 → RH. These observations support the hypothesis that air pollution contributes to climate change and that PM2.5 tends to exacerbate the urban heat island (Rybarczyk et al., 2025). Overall, our findings demonstrate that the causal relationship between PM2.5 and RH in Quito is both bidirectional and temporally dynamic. In the early 2000s, meteorological conditions predominantly influenced pollution levels. Conversely, by the early 2020s, air pollution began to exert a strong influence on the urban climate.

## 4 Discussion

This study addressed two research questions: (1) Does air pollution influence meteorological conditions? (2) Have the causal relationships between air pollution and meteorology changed over the past two decades? Drawing on a comparative analysis of four urban districts in Quito (Belisario, Cotocollao, Carapungo, and El Camal), it was shown that the causal dynamics between fine particulate matter (PM_2.5_) and relative humidity (RH) have shifted between two reference periods: the early 2000s and the early 2020s. By applying the Convergent Cross Mapping (CCM) nonlinear dynamic modeling, the results provide clear evidence of bidirectional causal relationships between PM_2.5_ and RH across all four districts (Figure 4). These relationships are both spatially and temporally variable, highlighting the importance of nonlinear, state-dependent methods for detecting complex atmospheric interactions (Chen et al., 2017; Rybarczyk et al., 2024). Our work is one of the rare demonstrations of bidirectional causality between air pollutants and meteorological factors. This finding is supported by a previous study demonstrating two-way causal interactions between PM_2.5_ and temperature RH in Quito, showing that meteorology not only affects pollution levels but is also influenced by pollution, forming feedback loops (Rybarczyk et al., 2025). The mutual causality between PM_2.5_ and land surface temperature (LST) suggests that urban heat island and air pollution dynamically reinforce each other. In a broader context, Chen et al. (2020) also described feedback mechanisms across China, where PM_2.5_ concentrations impact humidity, wind, and sunlight, which then feedback into pollution levels, particularly during stagnant winter conditions, indicating implicit bidirectional relationships.

### 4.1 Physical mechanisms

The effect of RH on PM_2.5_ is complex and inconsistent from one site to another. This variability was more pronounced in the past (early 2000s) than nowadays (early 2020s). It is perfectly illustrated by sites in the center and north of the city. For Belisario in 2004, the sign of the interaction fluctuated between−0.25 to 0.25. In the case of Carapungo and Cotocollao, RH led mainly to increased PM_2.5_ concentrations, consistent with hygroscopic growth mechanisms (Liu et al., 2018; Chen et al., 2019, 2022; Ge et al., 2024). On the contrary, the south part (El Camal) shows a positive effect of RH on PM_2.5_. This observation can be explained by the fact that RH can be associated with precipitation or scavenging processes that remove PM_2.5_ from the atmosphere, potentially reducing ambient PM_2.5_ during rain events (Zhou S. et al., 2025; Fujino and Miyamoto, 2022). A previous study on the impact of RH and precipitation on PM_2.5_ concentration in Quito aligns with this contrasted interaction (Zalakeviciute et al., 2018a). This work shows that the effect of the meteorological factors on the air pollutant depends on the pollution sources and humidity levels. A combination of high RH and low precipitations tend to increase pollution concentrations in busy traffic areas (e.g., Belisario), whereas the accumulation of fine particulate matter is primarily related to a lack of rain events in the industrial districts (e.g., Carapungo).

These findings reflect known but context-dependent atmospheric mechanisms. When humidity rises from low to moderate levels, it can enhance the formation of secondary aerosols through chemical reactions and cause existing particles to grow by absorbing moisture, both of which may contribute to increased PM_2.5_ concentrations. Several studies have shown that wintertime humidity exerted a strong positive influence on PM_2.5_ levels, especially under stagnant conditions (Zhou et al., 2015; Cheng et al., 2015; Qiu et al., 2015). However, at very high humidity levels (above 80%), particles may grow large enough to be removed from the air via wet deposition, reducing pollution (Wang and Ogawa, 2015; Zhang et al., 2015; Zalakeviciute et al., 2018a). For instance, Rahman and Meng (2024) found a strong negative correlation (*r* = −0.72) between relative humidity and PM_2.5_, suggesting that excessive humidity can lower pollution through removal processes.

By the early 2020s, the dominant direction of causality between PM_2.5_ and RH in Quito's districts had shifted markedly, with air pollution emerging as a significant driver of meteorological conditions. From the northern to the central part of the city, the PM_2.5_ → RH interaction strength has significantly increased from the early 2000s to the early 2020s (Figures 5a–c). Additionally, the sign of this interaction tends to become more negative, suggesting that fine particulate matter induces a reduction in atmospheric humidity. There is not a single universal mechanism, but several plausible pathways where air pollution can contribute to lower RH locally or under certain conditions. In Quito, at high altitude (about 2,850 m ASL) with complex orographic flow, the effect can be subtle and region-specific (Zalakeviciute et al., 2018a). There are at least three main lines of reasoning and physical processes involved. The first one is based on the aerosol-radiation interactions altering boundary-layer temperature and stability (Li et al., 2017; Huang et al., 2023). PM scatters and/or absorbs sunlight. This changes the surface radiation balance, typically reducing surface heating in polluted air (dimming) or, if absorbing aerosols (e.g., black carbon), warming the atmosphere aloft but cooling the surface. If surface cooling occurs or lower troposphere warms differently, the boundary layer can become more stable and shallower, reducing mixing and potentially lowering near-surface humidity through restricted moisture exchange with the free atmosphere. A shallower, more stable boundary layer can lead to drier near-surface air if moisture supply from above is limited, potentially lowering RH locally.

A second mechanism relies on the moisture transport and sources in the Andean context (Esquivel-Hernández et al., 2019; Bedoya-Soto and Poveda, 2024; Gutierrez-Villarreal et al., 2025). Quito sits in a narrow inter-Andean valley; upslope/downslope circulations (i.e., valley-mountain breeze) strongly modulate humidity. Pollutants can modify local cloud formation and precipitation processes, which in turn affect humidity distribution. For example, reduced low-level convection and altered cloudiness can decrease water vapor mixing into the boundary layer, depending on prevailing winds and stability. Polluted air can influence cloud formation via cloud condensation nuclei (CCN) and lifetime. If pollution suppresses shallow cumulus development or accelerates cloud evaporation, the local RH near the surface can decrease after clouds dissipate. The cloud microphysics and CCN process can also have another effect on the RH. Higher CCN from pollution can lead to more numerous but smaller cloud droplets, potentially affecting cloud cover and evaporation rates (Zhang et al., 2021; Eirund et al., 2022; Cheng et al., 2009). In daytime, this can lead to clearer skies but may also alter the balance between evaporation and condensation in the boundary layer, influencing RH profiles. If pollution promotes drizzle or rain in certain regimes, precipitation can remove moisture from the boundary layer, reducing RH, especially during or after rain events.

The last explanation is derived from the temperature-humidity coupling. RH is inversely related to temperature for a given water vapor content. If pollution leads to a temperature increase (e.g., through greenhouse-like effects in the urban canopy or aerosol indirect effects on radiation), RH can drop even if actual water vapor mixing ratio remains similar. Conversely, if pollution reduces surface temperature due to dimming, RH could rise. The observed reduction in RH would then require a scenario where temperature rises or moisture supply changes dominate. The causal impact of fine particulate matter on the urban heat island effect observed in a previous study supports the former hypothesis (Rybarczyk et al., 2025).

### 4.2 Spatial heterogeneity

Only El Camal exhibits a consistent pattern over the last two decades, which can be attributed to the fact that this district has the highest level of RH in the city (Figure 5d). Clouds from the Amazon rotate around the region's largest volcano (Cotopaxi, south of Quito), creating a humidity gradient that increases from north to south of Quito. Figure 1 shows that the dry area is expanding over the years. Our findings suggest that pollution is, at least partially, responsible for this expansion. The causal effect of PM_2.5_ on Quito's heat island supports this interpretation (Rybarczyk et al., 2025). This radiative mechanism is particularly evident in districts with higher traffic density and industrial activity, such as Belisario, Cotocollao, and Carapungo, which exhibit the most pronounced pollution-driven suppression of RH. Socioeconomic disparities further amplify these effects, with districts that have older fleets and fewer green spaces experiencing stronger impacts (Zalakeviciute et al., 2024).

### 4.3 Broader implications and policy relevance

Our findings confirm that the relationship between RH and PM_2.5_ is dynamic and bidirectional, which means that air pollutants have a stronger effect on meteorology than previously assumed. Traditionally, atmospheric models assume that meteorological variables influence air pollution levels, with little consideration for reverse or feedback effects. However, our results challenge this assumption. The CCM approach uncovers the causal effect of PM_2.5_ on RH. Such an outcome underscores the value of nonlinear analytical frameworks in revealing complex, state-dependent feedback that linear methods such as correlation often fail to detect (Istiana et al., 2023). The influence of PM_2.5_ on RH, once minor, has become significant over the last two-decades, worsening the dry climate affecting the north part of Quito. The dominance of this pathway in recent years signals a structural shift in the urban atmosphere, driven by emissions, urban topography, regulations, and climate change feedback. This underscores the urgent need to incorporate causality-focused, nonlinear methods into air quality assessments and policy planning, especially in rapidly changing, high-altitude urban environments.

An important outcome of this study is to emphasize that the relationship between air pollution and meteorology is increasingly shaped by human activity. Although this study did not perform chemical speciation of PM_2.5_, the observed shift toward pollution-driven RH suppression is consistent with well-documented mechanisms involving absorptive aerosols. Despite the various fuel regulations implemented over the last few decades (e.g., the adoption of Euro 3 fuel standards in 2017), the growth of the old vehicle fleet has led to an increase in black carbon (BC) emissions due to the incomplete combustion of diesel engines. BC is highly effective at absorbing solar radiation, which warms the atmosphere and reduces RH through enhanced evaporation and cloud suppression (Bond et al., 2013; Ramanathan and Carmichael, 2008; Wilcox et al., 2016). This interpretation, while not confirmed as the sole causal mechanism by this study's data, aligns with a growing body of international evidence documenting similar atmospheric transitions in major urban centers worldwide. In Beijing, sulfur reduction policies inadvertently increased black carbon concentrations (He et al., 2023). While the “China Clean Action Plan” significantly reduced PM_2.5_ levels, the decrease in sulfate aerosols may have altered atmospheric conditions, such as relative humidity and aerosol formation, indirectly raising black carbon concentrations and shifting the interaction between PM_2.5_ and meteorology. Additionally, Santiago de Chile has seen a strengthening of the causal relationship between air pollution and meteorology following the progressive implementation of stricter vehicle emission standards, including Euro 4 and higher, which altered emission profiles and enhanced interactions between PM_2.5_ and local climatic conditions (Feron et al., 2023; Tikoudis et al., 2022). These parallels suggest that Quito's experience is part of a broader global pattern of urban atmospheric evolution driven by anthropogenic emissions and policy shifts.

This reality demands more sophisticated and proactive policy interventions that move beyond conventional, linear assumptions. Based on our findings, specific, actionable recommendations include: (1) Integrating humidity and aerosol monitoring—particularly for absorptive species like black carbon—into urban air-quality early-warning systems to account for these newly understood feedback loops. (2) Shifting policy focus from single-pollutant or single-source regulations (like fuel standards alone) to integrated strategies that include fleet modernization and emissions controls targeting older vehicles, which are a key source of the BC driving the atmospheric shift. (3) Mandating the use of nonlinear dynamic models, like CCM, to pre-emptively assess proposed environmental regulations. This would help forecast potential unintended feedback—such as the trade-offs seen in Beijing or the muted benefits of fuel standards in Quito—before implementation, leading to more resilient and effective integrated air quality and climate strategies.

## 5 Conclusion and future work

This study applied Convergent Cross Mapping (CCM), a nonlinear empirical dynamic modeling technique, to investigate the causal relationship between fine particulate matter (PM_2.5_) and relative humidity (RH) in Quito, Ecuador. Using 20 years of hourly data from four urban districts across the city, the analysis revealed statistically significant, bidirectional causal relationships between PM_2.5_ and RH. These relationships were found to be nonlinear, state-dependent, and temporally variable.

In the early 2000s, RH exerted a dominant influence on PM_2.5_ concentrations, consistent with established atmospheric mechanisms such as hygroscopic growth and aqueous-phase chemistry. However, by the early 2020s, this dynamic had shifted, particularly in the industrial districts in the northern part of the city (e.g., Carapungo and Cotocollao), where PM_2.5_ emerged as the stronger causal driver, suppressing RH through radiative and microphysical mechanisms. This inversion was supported by partial derivative analyses, which show increasingly negative PM_2.5_ → RH interaction strength over time. These findings provide compelling evidence of a structural transformation in Quito's urban atmospheric system, driven by anthropogenic emissions—probably black carbon from factories and older diesel vehicles—and amplified by the city's high-altitude topography.

The results underscore the need for integrated air quality and climate policies that account for the bidirectional and evolving nature of pollution-meteorology interactions. Regulatory interventions, such as fuel standard upgrades, must consider their potential to alter pollutant composition and trigger unintended feedback. In Quito, the shift toward PM_2.5_-driven RH suppression could plausibly be linked to a relative increase in black carbon-rich PM_2.5_ components, potentially influenced by factors like the implementation of Euro 3 fuel standards alongside an aging vehicle fleet. This exemplifies a potential trade-off where policies designed to address one aspect of air quality might inadvertently influence others. Urban planning strategies should therefore prioritize fleet modernization, green infrastructure, and emissions monitoring to mitigate pollution-driven climate effects.

Future research should expand the scope of analysis to include additional meteorological variables (e.g., temperature, wind speed) and pollutants (e.g., BC, PM_10_, NO_2_, O3) to capture a more comprehensive picture of urban atmospheric dynamics. Specifically, extending CCM to include temperature and black carbon in the districts showing strong PM_2.5_-driven RH reduction would be crucial to directly verify the hypothesized aerosol-radiation and temperature-humidity coupling pathways. Also, longer and additional time windows (e.g., early 2010) could offer deeper insights into intermediate and long-term trends that may be useful for modeling future predictive scenarios. Methodological advancements in parameter selection and attractor reconstruction would further enhance the reliability of CCM applications. Finally, comparative studies across cities with diverse topographies and emission profiles would also help generalize findings and inform global urban climate resilience strategies.

## Data Availability

Publicly available datasets were analyzed in this study. This data can be found here: https://aireambiente.quito.gob.ec/.

## Appendix B

**Table A.1:** Mean cross-map skills for the four districts and two periods.

**District**	**Year**	**RH → PM_2.5_ (*ρ*)**	**PM_2.5_ → RH (*ρ*)**
Belisario	2004	0.33	0.20
	2024	0.37	0.14
Carapungo	2005	0.45	0.49
	2024	0.21	0.37
Cotocollao	2005	0.38	0.26
	2024	0.26	0.15
Camal	2004	0.32	0.48
	2022	0.31	0.39
